# A Comparison of Eye Tracking Latencies Among Several Commercial Head-Mounted Displays

**DOI:** 10.1177/2041669520983338

**Published:** 2021-02-11

**Authors:** Niklas Stein, Diederick C. Niehorster, Tamara Watson, Frank Steinicke, Katharina Rifai, Siegfried Wahl, Markus Lappe

**Affiliations:** Institute for Psychology, University of Muenster, Muenster, Germany; Lund University Humanities Lab and Department of Psychology, Lund University, Lund, Sweden; School of Social Sciences and Psychology, Western Sydney University, Penrith, Australia; Department of Informatics, University of Hamburg, Hamburg, Germany; ZEISS Vision Science Lab, Carl Zeiss Vision International GmbH, Tübingen, Germany; Institute for Psychology, University of Muenster, Muenster, Germany; Otto-Creutzfeldt Center for Cognitive and Behavioural Neuroscience, University of Muenster, Muenster, Germany

**Keywords:** virtual reality, eye tracking, gaze-contingent rendering

## Abstract

A number of virtual reality head-mounted displays (HMDs) with integrated eye trackers have recently become commercially available. If their eye tracking latency is low and reliable enough for gaze-contingent rendering, this may open up many interesting opportunities for researchers. We measured eye tracking latencies for the Fove-0, the Varjo VR-1, and the High Tech Computer Corporation (HTC) Vive Pro Eye using simultaneous electrooculography measurements. We determined the time from the occurrence of an eye position change to its availability as a data sample from the eye tracker (delay) and the time from an eye position change to the earliest possible change of the display content (latency). For each test and each device, participants performed 60 saccades between two targets 20° of visual angle apart. The targets were continuously visible in the HMD, and the saccades were instructed by an auditory cue. Data collection and eye tracking calibration were done using the recommended scripts for each device in Unity3D. The Vive Pro Eye was recorded twice, once using the SteamVR SDK and once using the Tobii XR SDK. Our results show clear differences between the HMDs. Delays ranged from 15 ms to 52 ms, and the latencies ranged from 45 ms to 81 ms. The Fove-0 appears to be the fastest device and best suited for gaze-contingent rendering.

## Introduction

In the past decade, the rapid commercialization of virtual reality (VR) equipment has made head-mounted displays (HMDs) available to a much larger number of researchers. Head position tracking is included in most devices and has opened interesting avenues to study perception with naturally moving observers in small areas (e.g., Bansal et al., 2019; Niehorster et al., 2017; Scarfe & Glennerster, 2015; Warren et al., 2001; Wexler & van Boxtel, 2005). Now, the integration of eye trackers in HMDs promises to be the next technological step, allowing even more immersion in virtual environments (VEs).

Since the first objective measurement of eye movements by Delabarre (1898) and Huey (1898), eye tracking techniques have come a long way (Wade, 2010). In the 1950s, the first contact lenses including coils were used to precisely record where a subject is looking (Holmqvist et al., 2011). Electrooculography (EOG), a less invasive method, was the most applied method in the 1970s (Young & Sheena, 1975). By measuring the changes of the electric field evoked by changes of the position of the pigmented epithelium using electrodes placed around the eye, one can determine the eye ball orientation with respect to the head position. Although the data can be collected at high sampling rates, EOG lacks spatial accuracy, especially on the vertical axis (Duchowski, 2007). Thus, today’s most used method is video-based eye tracking, combining a noninvasive method with sampling rates up to 2000 Hz and an advertised spatial accuracy down to 0.15° (SR Research, 2020). Progress in camera technology made it possible to create head-mounted eye trackers (including cameras filming the wearer’s field of view [FOV]) that estimate eye positions in the real world without explicitly recording head movements (e.g., Niehorster Hessels, et al., 2020; Niehorster, Santini, et al., 2020). The technology is now included in HMDs, promising advances in wearing comfort by software that helps the user to adjust the position of the HMD on the head to optimize optical parameters to the location of the eye balls. Moreover, it allows analyzing the location and length of fixations in VEs and the implementation of new interaction methods (Piumsomboon et al., 2017).

Now that eye trackers in HMDs have become available from multiple vendors, we may ask for what kinds of application or experiment they are suitable for. From their low sampling rate (generally around 100 Hz), it is clear that these eye trackers cannot be used for analyses of trial by trial saccade dynamics (Mack et al., 2017), although it should be noted that successful attempts have been made to determine saccade dynamics through a modeling approach using low sampling rate and low noise data, when averaging over many saccades (Gibaldi & Sabatini, 2020; Wierts et al., 2008). It remains an open question whether these methods would work reliably on the off-line data provided by the eye trackers examined in the current study.

In contrast, for the study of looking behavior by means of recording fixations (periods during which the image of an object is held stable on the retina; see Hessels et al., 2018), the lower sampling rates do not pose significant issues (Andersson et al., 2010). For measures based on fixations, the important factors are accuracy and precision (Niehorster, Zemblys, et al., 2020), which may affect the minimum size of objects to which gaze can be reliably resolved (Hessels et al., 2016, 2017; Holmqvist et al., 2012). Assessment of these aspects of eye trackers in HMDs is thus an important topic for those types of applications (e.g., Adhanom et al., 2020).

In the present study, we are concerned with a third type of application, gaze-contingent display paradigms in perceptual research. Gaze-contingent displays rely on low-latency access to eye tracking data and the ability to rapidly adapt the visual stimulation presented in the HMD in response to an eye movement. In such conditions, a host of perceptual effects related to eye movements can be studied and used in applications: Examples include, first, blinks which can be detected (Alsaeedi & Wloka, 2019) and used to apply nonperceivable modifications to a VE (Langbehn et al., 2018). Second, saccadic omission, often also called saccadic suppression, refers to the phenomenon that stimuli presented during a saccade are often not consciously perceived (Campbell & Wurtz, 1978; Diamond et al., 2000; Holt, 1903). This includes the self-generated motion on the retina that occurs as the eye sweeps across the scene during the saccade (Ilg & Hoffmann, 1993). Saccadic suppression of displacement refers to the failure to perceive a displacement of a persistent stimulus in the scene during the saccade, or even the saccade target itself (Bridgeman et al., 1975). Change blindness refers to the failure to notice changes to objects in the scene, including their color or position, as well as the introduction or removal of objects (Rensink et al., 1997; Simons & Levin, 1997). In addition, there are also several phenomena of changes to the perceived position of objects briefly presented at the time of a saccade (Lappe et al., 2000; Matin & Pearce, 1965; Ross et al., 1997). Next to the spatial aspects of scene vision, there are also changes to temporal processing associated with saccades. Chronostasis, that is, the stopped clock illusion, refers to an illusion in which time appears to briefly halt during the saccade (Yarrow et al., 2001), likely because of deployment of attention toward the saccade target (Georg & Lappe, 2007). Moreover, temporal intervals are misperceived, and the temporal order of successively presented stimuli may even be reversed (Morrone et al., 2005).

These perceptual phenomena are commonly studied with gaze-contingent experimental paradigms. The ability to use such paradigms with HMDs in VR would open up many new research avenues with more naturalistic settings, for example, in the combination of eye and head movements (Anderson et al., 2020). Moreover, perceptual phenomena can also be applied to enhance VR scenarios (Bolte & Lappe, 2015; Bruder et al., 2013; Sun et al., 2018). There are several applications that make use of these techniques, for example, environments with artificial scotoma (Bertera, 1988) and gaze-contingent foveated rendering techniques (Patney et al., 2016). In gaze-contingent rendering, the resolution of the rendered image can be imperceptibly reduced in the periphery because peripheral visual resolution is much lower than foveal visual resolution. Using eye tracking, the location of a high resolution area in the image can be updated according to gaze direction.

Besides perception, another common use of gaze-contingent paradigms is the study of calibration of motoric functions of the brain, involving stimuli that change during saccades. Changes applied during a saccade can have long-lasting effects mediated by implicit learning. Consistently applied displacement of the saccade target lead to adjustments in saccade amplitude known as saccadic adaptation (McLaughlin, 1967; Pélisson et al., 2010). These adjustments can be specific for different depth planes in the three-dimensional space (Chaturvedi & Van Gisbergen, 1997). Moreover, recalibration can also occur in opposite directions in each eye at the same time, suggesting the existence of independent monoculomotor and binoculomotor plasticities for each eye (Maiello et al., 2016). Saccadic adaptation involves not only motor learning but also persistent changes in perception of visual space (Collins et al., 2007; Zimmermann & Lappe, 2016). Trans-saccadic changes of the size of a target object lead to a recalibration of size perception in peripheral vision (Bosco et al., 2015; Valsecchi & Gegenfurtner, 2016) and to a recalibration of grasping action (Bosco et al., 2015). Also, peripheral shape or color perception can be modified by trans-saccadic changes (Herwig & Schneider, 2014; Paeye et al., 2018).

For all of these applications, correct timing of the respective stimulation or display change in relation to the saccade is essential. These phenomena are transient and occur typically only up to the end of the saccade or some few tens of milliseconds thereafter, often with a common time course (Burr et al., 2010; Ross et al., 2001). For example, saccadic suppression fades out by the end of the saccade (Diamond et al., 2000), as do possible impacts on localization or timing. Saccadic suppression of displacement occurs if the displaced stimulus is visible at the end of the saccade but not if it is instead presented 100 ms later (Deubel et al., 1996). Saccadic adaptation also works best if the displaced target is present at saccade onset and less well if it is delayed (Fujita et al., 2002). From the time courses of these effects, it is clear that effective methods for gaze-contingent displays for vision research, although dependent on the specific paradigm, should ideally be made with stimulus delays that do not exceed the duration of the saccade by more than a few tens of milliseconds (e.g., Loschky & McConkie, 2000). Similar requirements exist for foveated rendering in VR (Albert et al., 2017).

In the present study, we evaluated eye tracking latency of currently available HMDs to investigate their suitability for gaze-contingent display paradigms. We recorded delays of eye trackers in three commercially available HMDs and compared them to simultaneously recorded EOG. As a current gold standard for comparison, we perform the same analysis on a stationary, screen-based eye tracker (Eyelink 1000) that is well established in the vision research community.

## Setup 

In this section, we describe the hardware, software, and calibration method for each setup, including versions and configurations. All recordings were done on the same computer with an Intel Core i9 9900K (3.6 GHz) processor, 8 GB RAM, and a NVIDIA RTX2080 graphics card (Driver Version 26.21.14.3086). The operating system was Windows 10 (Version 1809, Build 17763.914) with the NVIDIA driver set to maximum performance and minimum latency mode. To obtain consistent time stamps, we implemented and compiled a CPU-based high-precision clock script that could be used across all software packages. In all tests, we used the first time stamp after a sample was available to the recording software (Unity3D, 2019.1.8f1). Recordings were made using the following devices:

### Head-Mounted Displays

#### Fove-0

The Fove-0 was the first commercially available HMD that included eye tracking. The first devices were delivered in January 2017 and included a camera-based eye tracking system with an advertised spatial tracking accuracy of less than 1°. The maximum eye tracking sampling rate is 120 Hz. The display has a refresh rate of 70 Hz (Fove Inc., 2017). We used it with the FoveInterface prefab of the Fove-0 Unity package (Version 0.16). This approach reduces the rate of eye tracking samples to 70 Hz.

Although this approach does not use the full 120 Hz capability of the eye tracker, the method of synchronizing to the display frame rate allows to receive the most recent data point at the start of each frame and to manipulate the next frame accordingly. Access to data at higher sampling rates would not allow to change the effective latency of the whole system, as the manipulation of the visual stimuli can only be done frame by frame. Unity’s XR settings were set to Stereo Display.

#### Varjo VR-1

The Varjo VR-1 was introduced in October 2019 and includes a secondary (foveal) display to provide a higher resolution in the center of the main 87° FOV display. The eye tracker samples at 100 Hz, and the display has a refresh rate of 90 Hz (Varjo Technologies, 2019). Our recordings used Unity3D (2019.1.8f1), the Varjo Base Software (Version 2.2.1.17), and SteamVR. The Varjo API provides for each frame a list with one or two eye data samples. We always used the most recent sample to manipulate the next frame. Unity’s XR settings were set to OpenVR.

#### Vive Pro Eye

The Vive Pro Eye was introduced in June 2019 and is an upgraded version of High Tech Computer Corporation (HTCs) Vive Pro that includes the Tobii eye tracking system that is also present in other headsets (e.g., Pico Neo 2 Eye and Tobii HTC Vive Devkit). The headset has a declared eye tracking accuracy of 0.5° to 1.1° at 120 Hz, a 110° FOV, and a display with a refresh rate of 90 Hz (HTC, 2019). We used it with Unity3D (2019.1.8f1), SRanipal (v1.1.0.1), and SteamVR. Unity’s XR settings were set to OpenVR.

### Comparison Devices

#### EOG

**Figure 1.**
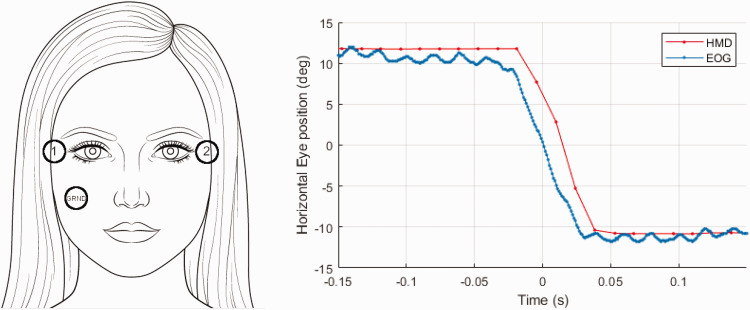
EOG. Left: Placement of the electrodes to record the electronic signal. Right: Shifted and aligned EOG and HMD signals, where the EOG crossed 0° is defined as 0 ms. HMD = head-mounted display; EOG = electrooculography.

For EOG recordings simultaneously to data collection from the HMD eye trackers, we used the same setup as described in (Bolte & Lappe, 2015). It consisted of a BioVision EOG amplifier (BioVision, Germany) used with two self-adhesive electrodes next to each eye and a third electrode as ground on the left cheek (see Figure 1). Amplified EOG signals were read in via analogue ports of a Meilhaus RedLab-1208FS PLUS sampling device. Because the pigmented epithelium in the retina evokes changes of an electric field when saccades are made, the difference between the electrodes next to the subject’s eyes provides zero-latency signals of horizontal eye movement. Our setup collected EOG data at sampling rates of approx. 800 Hz. Sampled EOG data were recorded in Matlab R2018b and Psychtoolbox Version 3.0.14 concurrently with the HMD’s eye tracking data.

#### Eyelink 1000

For comparison to the HMD eye trackers, we included the Eyelink 1000 from SR Research as a baseline device for its high temporal resolution (1000 Hz) and low latency. It has become a gold standard of eye tracking research in the past decades and is extensively used in latency critical experiments. The stationary eye tracker was connected via an Intel onboard ethernet port (I219-V). Stimuli were presented on a FlexScan 930 screen from EIZO with a resolution of 1,280 × 1,024 pixels. Movements of the head were minimized by using a chin rest and a headrest, allowing a declared latency of below 1.8 ms (SR Research Ltd., 2009). The device was set to monocular recording to achieve the highest possible sampling rate (1000 Hz).

## Study 1: Eye Tracking Delay

In this study, we measured the time it takes for the HMD’s eye tracker to register a change in eye position and deliver this information to the experimental software. We call this the delay of the eye tracker. We measure it by registering the same eye movement in EOG and HMD and calculate the time difference between the two signals.

### Methods

#### Participants

We measured all setups with four participants (3 male, 1 author, all employees of the University of Muenster, normal vision, no contact lenses, no glasses, all experienced with eye tracking experiments, age 25–46). Informed, written consent was obtained from all participants.

#### Procedures

In four sessions, that is, one for each of the HMDs (Eyelink 1000, Fove-0, Varjo VR-1, Vive Pro Eye), the participants made 60 horizontal saccades of 20° in between two persistently visible targets from left to right and vice versa. The start of all trials was indicated by an auditory cue, which was played alternating between 0.5s and 1s after the start of the previous trial. Participants were instructed to fixate a target and make a saccade to the opposite target after an auditory cue.

#### Data Analysis

For the measurement of the delay of the HMD eye tracker, we matched saccade data from the EOG and the HMD (only left eye used) and determined the temporal offset between them (Figure 1). The EOG signal, which is recorded as a voltage, was scaled for each saccade to match the repeated gaze direction at start and end points of the matching saccade from the HMD tracker, provided in degrees of visual angle. Then, the point in time at which the eye position computed from the EOG signal crossed the center of the screen (0°) was taken as the reference time point (0 ms; see Figure 1). Next, data samples (time stamps and eye positions) were averaged, separately for EOG and HMD data, across all valid saccades to derive a mean saccade time course for each subject. Saccades smaller than 7.5° were discarded (4.5% of all saccades, see Table 1). All saccades were manually checked to ascertain their validity.

**Table 1.** Overview of All Results.

	Fove-0	Varjo VR-1	Vive Pro Eye	Tobii XR	Eyelink 1000
Study 1					
Eye tracking delay (ms)	15 (2.5)	36 (3.3)	52 (3.2)	–	0 (3.4)
Number of saccades	236 (98.33%)	218 (90.83%)	232 (96.66%)	–	231 (96.25%)
Sampling rate	70.1	99.1	91.8	–	1000
Study 2					
Eye tracking delay (ms)	16 (1.3)	36 (2.7)	50 (1.8)	51 (1.2)	–
End-to-end latency (ms)	45 (6.1)	57 (6.6)	79 (5.4)	81 (2.6)	–
Display delay (ms)	29	21	29	30	–
Number of saccades	177 (98.33%)	170 (94.44%)	171 (95%)	174 (96.66%)	–
Sampling rate	69.7	98.1	88.3	82.5	–

*Note*. Standard deviations and % of planned saccades are shown in brackets. The analysis includes only saccades that could be detected in both the optical eye tracker and the EOG. The EOG achieved a mean sampling rate of 805 Hz (Study 1) and 1196 Hz (Study 2). Display delay in Study 2 was determined as the difference between end-to-end latency and eye-tracking delay for each device. The display delay can include things such as rendering time, transmission time of the signal, and latency of the display.

Finally, to calculate the tracker delay, time lags of HMD samples of eye positions during the saccade (i.e., within a range from 20% to 80% of the mean saccade amplitude) were taken relative to the same eye position samples of the EOG.

### Results

**Figure 2.**
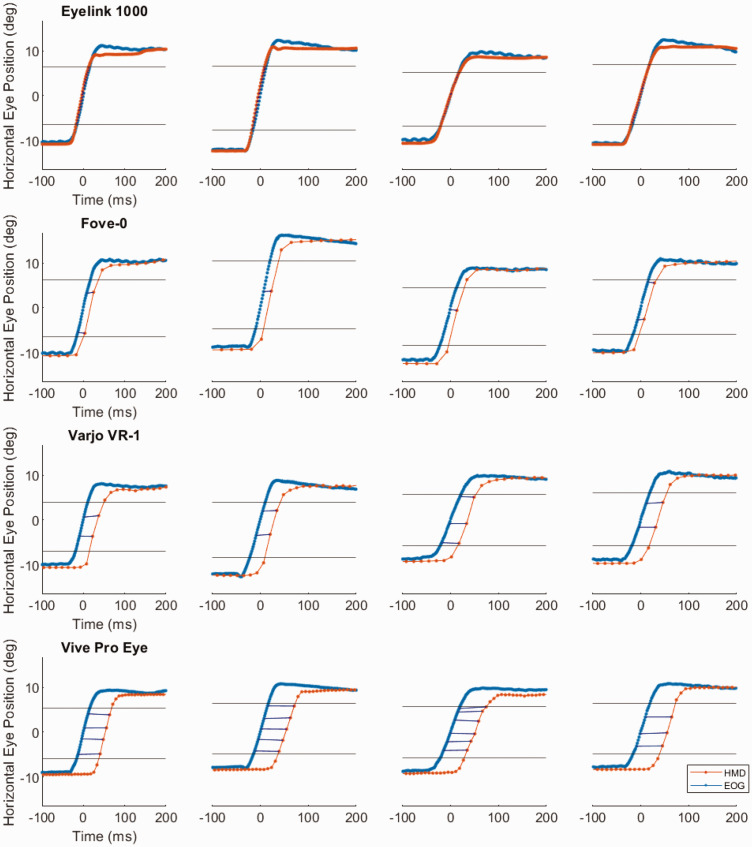
Rightward mean saccades. Averaged saccades recorded with the HMDs (red) and the EOG (blue) of all four participants. Eye tracking delays are included as blue lines. Horizontal black lines show 20% to 80% of the mean amplitude. HMD = head-mounted display; EOG = electrooculography.

**Figure 3.**
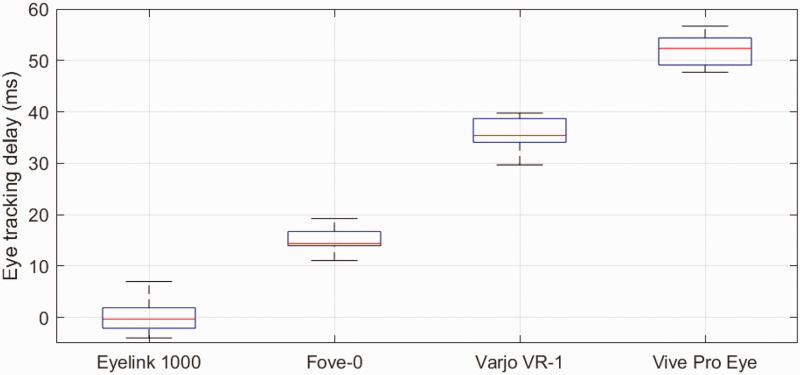
Average eye tracking delays of all tested HMDs in comparison to the EOG. Measurements of the Eyelink 1000 are included as a baseline. The digitization of the EOG signal leads to some delay. The Eyelink 1000 has an advertised latency of below 2 ms. Since EOG and Eyelink have a relative delay of 0 ms, the EOG has a similar low delay.

Figure 2 shows the averaged eye positions during saccades for each subject in each of the devices. The comparison between the Eyelink 1000 and the EOG shows no delay between the two methods (0 ms). Among the three HMDs, the Fove-0 shows the lowest delay (15 ms), followed by the Varjo VR-1 (36 ms) and the Vive Pro Eye (52 ms). Figure 3 gives an overview of averaged rightward saccades of all the devices.

A linear model was fitted to predict the *mean delay* between each eye tracker and the EOG using *device* and *saccade direction* as factors and the Eyelink data as baseline (*adjusted R*^2^ = .98, *RMSE* = 0.0031, *df* = 3, 28). All devices showed significantly larger delays than the Eyelink baseline. Tukey–Kramer-corrected post hoc *t* tests revealed significant differences between all device pairs (*p* <.001). There was no significant difference between saccade directions.

## Study 2: Latency for Saccade-Contingent Display Update

If the HMD is used as a gaze-contingent display, the screen needs to be updated as soon as possible after the onset of a saccade. The latency of saccade-contingent update of the HMD screen depends on at least two further factors in addition to the tracker delay. The first is the speed of the detection of the saccade from the eye tracking data, which varies with the algorithm used but may also depend on the sampling frequency. The second is the time it takes to update the screen, which depends on rendering time, frame rate, and video hardware. In addition, communication standards and bus architectures, as well as hard- and software used for transmitting eye tracker and video signals between the HMD and the computer, can add latency. We aimed to compare the end-to-end latency of saccade-contingent display update between the HMDs, using in each case the same saccade detection algorithm and the same video hardware, to measure the end-to-end latency in comparable minimum latency conditions. For this purpose, we used a simple Unity script that changed the background color of the VE in the HMD from white to black as soon as the eye position samples crossed the midline between the two targets (i.e., 0°). The black background was then present for the next 50 frames before it switched back to white for the next trial. The prerendering setting in Unity was turned off. 

### Methods

#### Setup

**Figure 4.**
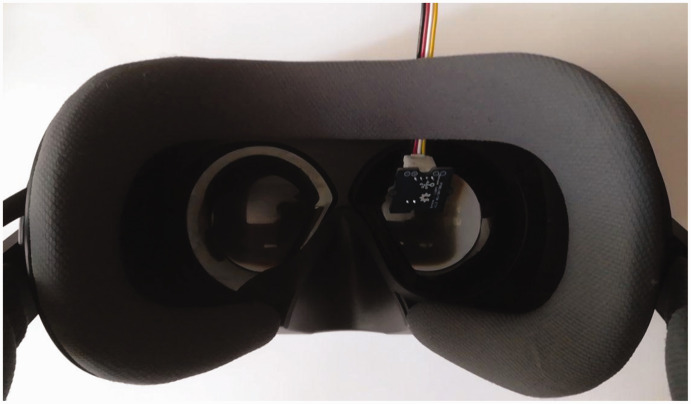
Varjo VR-1 with photodiode placed on the right screen.

The setup was the same as described earlier using the same HMDs. To record the time of the display change, a photodiode was fixed to the right display of each HMD (see example in Figure 4). The photodiode was connected to an analogue input port of a RedLab-1208FS PLUS that was used to record the EOG signals. To prevent any sampling problems, we increased the maximum recording frequency of the input ports to 1500 Hz.

Furthermore, we measured the Vive Pro Eye twice in this experiment, once with the SRanipal SDK provided by the device as before and, second, with the Tobii XR SDK. We included this second measurement with a different SDK in the hope that the Tobii XR SDK might produce shorter delays than the native SDK.

#### Participants

We performed all measurements with three participants (2 male, 1 author, no contact lenses, no glasses, age 20–26, all experienced with eye tracking experiments). Informed, written consent was obtained from all participants.

#### Procedures

The procedures were the same as in Study 1. In addition, when the eye tracker from the left eye returned a gaze position that crossed the vertical midline of the FOV, the background color of the VE in the HMD changed from white to black. To determine the time at which the change of the display from white to black occurred, we applied a change point detection algorithm (Killick et al., 2012) on the sampled photodiode signal. In case of multiple candidates, we used the last change point before the screen became and stayed black. Trials in which EOG saccades did not trigger a photodiode change in a time window of (0 + 200 ms) were excluded. All saccades were then manually checked to ascertain their validity. Overall, 692 of the 720 saccades (96.11%) were included in the analysis (see Table 1 for an overview). From this data, we calculated mean saccades for each subject, device, and direction, as before.

### Results

**Figure 5.**
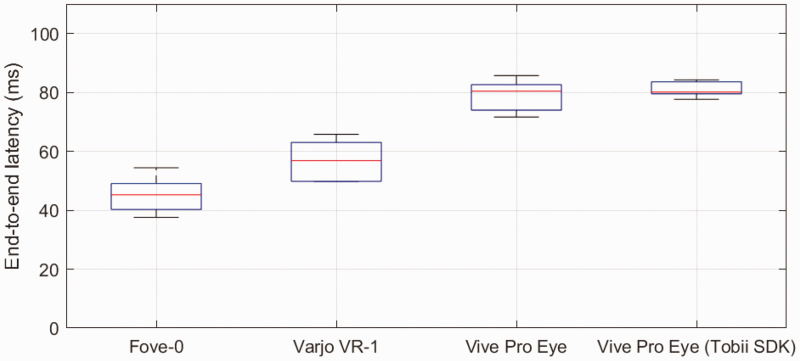
Average saccade-contingent latency of all tested HMD eye trackers in comparison to the EOG. End-to-end latencies of all tested HMD eye trackers based on the EOG signal and a photodiode capturing changes on the HMD display.

Figure 5 shows average end-to-end latencies for the tested devices. For each mean saccade, we calculated the mean end-to-end latency of saccade-contingent display update from the time difference between the middle of the saccade in the EOG signal and the time of display change determined from the photodiode signal. Among the four HMDs, the Fove-0 showed the lowest latency (45 ms), followed by the Varjo VR-1 (57 ms), the Vive Pro Eye with the native SDK (79 ms), and the Vive Pro Eye using the Tobii XR SDK (80 ms). Using the resulting 24 mean latencies for each subject, direction, and device, an analysis of variance showed a significant difference between devices (*F* = 62, *p* <.001, *df* = 3, 20). Moreover, Tukey–Kramer-corrected post hoc *t* tests revealed significant differences between the latencies of all devices except for the two conditions using the Vive Pro Eye (Fove-0 compared with Varjo VR-1 *p* <.01, all others *p* <.001).

**Figure 6.**
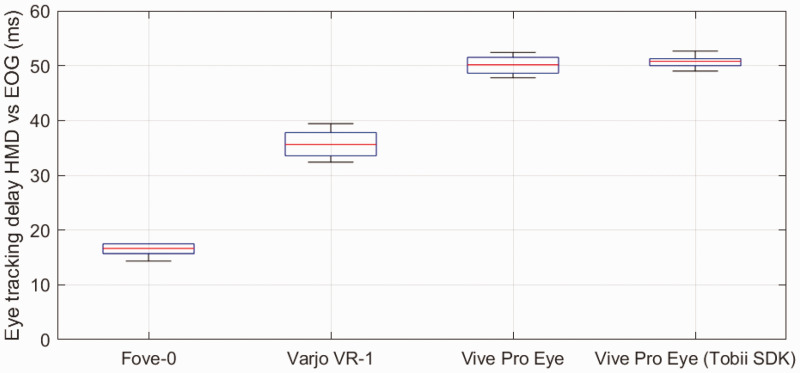
Average eye tracking delays of all tested HMD eye trackers in comparison to the EOG. The eye tracking delays of the second test confirm previous results. The Tobii XR SDK did not make a difference for the delays of the Vive Pro Eye. HMD = head-mounted display; EOG = electrooculography.

Because the setup and procedure was largely the same as in the prior measurement of tracker delay, we also evaluated tracker delay in this measurement series to confirm previous results in the separate delay measurements of Test 1. Figure 6 shows tracker delay in this measurement series. Using the resulting 24 mean saccades with delays for each subject, direction, and device, an analysis of variance revealed significant differences for devices (*F* = 454, *p* <.001, *df* = 3, 20), but not saccade direction. Moreover, Tukey–Kramer-corrected post hoc *t* tests revealed significant differences between the delays of all device conditions (*p* <.001), except for the two conditions using the Vive Pro Eye, which were not significantly different from each other. Display latency was determined by subtracting end-to-end latency from eye tracking delay (see Table 1).

## Discussion

We measured tracker delays and end-to-end latencies of three HMD eye trackers in comparison to EOG. The Fove-0 had the lowest tracker delay in both tests (15 and 16 ms) and an end-to-end latency of 45 ms. The Varjo VR-1 had an eye tracking delay of 36 and 35 ms and an end-to-end latency of 57 ms. The Vive Pro Eye, either with its native SDK or with the Tobii SDK, was considerably slower with tracker delay of 50 ms and an end-to-end latency of 80 ms. The values of the Vive are out of the range suitable for gaze-contingent experiments (Loschky & McConkie, 2000). The latencies achieved by the Fove-0 or the Varjo VR-1 appear more suitable to such tasks (Loschky & Wolverton, 2007).

We do not know what causes the differences in tracker delay between the devices. One possibility, other than hardware differences, might be extensive low-pass filtering of the eye position data in the SDK. Such filtering reduces spatial noise (which might allow more accurate gaze position estimation) at the cost of latency. It may thus be that different manufactures put different emphasis on spatial versus temporal accuracy for different use cases. Indeed, we noticed informally that a recent software update (Version 0.17) of the Fove-0 SDK, which was advertised to improve spatial accuracy, produced longer delays than the ones we measured here. Any trade-off between spatial and temporal accuracy would be different for different research applications. Because using an HMD as gaze-contingent display has different requirements regarding spatial filtering and latency than using it for the analysis of fixation distribution, for researchers and developers, it is highly desirable to get full access to filtering and raw data from each eye tracking sample with minimal delay.

Our measurements of delay and latency were taken in comparison to EOG. The EOG operates in principle without any delay because it measures changes in electrical voltage directly produced by the change in orientation of the eyeball. However, the digitization of the EOG signal adds a small delay also to the EOG data. For accurate delay estimates of the HMDs, the delay introduced by the EOG needs to be taken into account. We cannot truly measure this delay, but based on the sampling rates we used, it should be on the order of 1 or 2 ms. This is consistent with the delay measured between the EOG and the Eyelink, our reference system, which came to a mean of 0 ms. As the Eyelink is expected to have a delay of 1.8 ms (SR Research Ltd., 2009), the delay of the EOG sampling should be in the same range. Given the size of the measured effects and the assumption that the delay of the EOG should be the same for all HMDs, this does not affect our results.

In placing the photodiode for the measurement of end-to-end latency, we aimed for similar layouts of the photodiode in each subject and device. However, there is some variance resulting from different head shapes of the subjects who took part in the study. Moreover, the pixel matrix of different HMDs might be refreshed with timing differences between the first and the last pixel. However, this could create at maximum a difference between two systems of one inter frame interval (e.g., 11 ms at 90 Hz) and thus cannot explain the differences we found between devices.

All three of our tested HMDs show rather large end-to-end latencies when compared with eye tracking devices currently used in research (e.g., Gibaldi et al., 2017). However, applications using off-line analysis of fixations in VR can be done with all devices. In this context, considerations regarding spatial resolution and data quality are more important than online latency. Off-line analysis of fixations, even if time-critical, seems to be possible with all HMDs as the delays and latencies had small variance and thus were quite stable. However, any analyses of saccadic reaction times or times of first fixation, and so forth will have a constant bias due to the eye tracking delay. Researchers interested in such measures should therefore measure the delay in their setup and correct for it in their analysis.

Based on our results, it appears that the Fove-0 and the Varjo VR-1 can be used as gaze-contingent display to a certain extend. However, it is important to note that for our measurement of end-to-end latency for gaze-contingent display change, we used a rather large (20°) saccade and a very simple detection algorithm (crossing of the screen center). These choices were made to allow a robust and comparable measurement of delay. Gaze-contingent research and applications are likely to involve different saccade amplitudes and more complex detection algorithms. Saccade amplitudes are highly dependent on task, for example, when looking at a natural environment the typical saccade varies between 2° and 20° (Bahill et al., 1975; Gibaldi & Banks, 2019; Land et al., 1999; McConkie et al., 1984; Sprague et al., 2015). Smaller saccades are more difficult to detect than larger saccades. Moreover, smaller saccades are also shorter in duration and might last for only 30 ms or much less. This might reduce the applicability of the tested HMDs for gaze-contingent research with small saccades. Therefore, researchers should do their own tests for their specific setup and scenario to examine whether the HMD eye tracker is suitable for their needs.

A further issue for online saccade detection concerns the sampling rate of the eye tracker. Reliable onset detection can be hard using sampling rates of the tested systems (70–90 Hz). The eye trackers in our test HMDs are capable of higher sampling rates than frame rates, which might be helpful for implementing reliable saccade detection algorithms. Moreover, more advanced methods might allow to achieve lower latency saccade onset detection (e.g., Arabadzhiyska et al., 2017). Still, as eye tracking in HMDs may become more advanced in the near future, access to faster eye tracking data with lower delay and lower end-to-end latencies would constitute an important step for VR and perception research.
